# Supplementary material for: Matching sensor ontologies through siamese neural networks without using reference alignment
Source: PeerJ Comput Sci. 2021 Jun 18;7:e602. doi: 10.7717/peerj-cs.602 (PMC8237319; doi:10.7717/peerj-cs.602)
Supplement: Supplemental Information 1 [file peerj-cs-07-602-s001.zip › 250-6/onto.html]

Author: Nick Knouf <nknouf@mit.edu>  
Contributor: Antoine Zimmermann <antoine.zimmermann@inrialpes.fr>, Jérôme Euzenat,   
Date: 08/06/2005  
Version: $Id: onto.rdf,v 1.30 2008/05/27 14:41:13 euzenat Exp $

## Classes

**http://www.w3.org/1999/02/22-rdf-syntax-ns#List** (, *)*


**http://xmlns.com/foaf/0.1/Person** (, *)*


**http://xmlns.com/foaf/0.1/Organization** (, *)*


**sqdsq** (, *)*
:   **Book** (, *)*
    :   **dzajndsq** (, *)*


        **sqxsqkd** (, *)*


        **zdqssqdb** (, *)*

    **qsdsnbsqd** (, *)*
    :   **Booklet** (, *)*


        **LectureNotes** (, *)*


        **dcsqdsq** (, *)*


        **Unpublished** (, *)*

    **Part** (, *)*
    :   **Article** (, *)*


        **dzqndbzq** (, *)*


        **vccfsq** (, *)*


        **dcsqdcsqd** (, *)*


        **InProceedings** (, *)*

    **zdazsx** (, *)*
    :   **xsqlknk** (, *)*


        **PhdThesis** (, *)*

    **Misc** (, *)*


    **dqzdxdcsqj** (, *)*
    :   **sdcsqhyz** (, *)*


        **Deliverable** (, *)*

    **dscdscg** (, *)*

**qsdsquj** (, *)*


**zqedzbx** (, *)*


**Address** (, *)*


**Institution** (, *)*
:   super: *http://xmlns.com/foaf/0.1/Organization*  


    **zauio** (, *)*


    **zadazxn** (, *)*

**dsqdbz** (, *)*
:   super: *http://www.w3.org/1999/02/22-rdf-syntax-ns#List*

**PageRange** (, *)*


**Date** (, *)*

## Properties

**http://www.w3.org/1999/02/22-rdf-syntax-ns#first**: http://www.w3.org/1999/02/22-rdf-syntax-ns#List -> \_ *()*


**http://www.w3.org/1999/02/22-rdf-syntax-ns#rest**: http://www.w3.org/1999/02/22-rdf-syntax-ns#List -> http://www.w3.org/1999/02/22-rdf-syntax-ns#List *()*

**http://purl.org/dc/elements/1.1/creator**\_ -> \_ *()*


**http://purl.org/dc/elements/1.1/contributor**\_ -> \_ *()*


**http://purl.org/dc/elements/1.1/description**\_ -> \_ *()*


**http://purl.org/dc/elements/1.1/date**\_ -> \_ *()*


**http://xmlns.com/foaf/0.1/firstName**\_ -> \_ *()*


**http://xmlns.com/foaf/0.1/name**\_ -> \_ *()*

## Individuals

<rdf:List@ttp://www.w3.org/1999/02/22-rdf-syntax-ns#nil>


<foaf:Person@a04570373>
:   - foaf:name = 'John-Jules Meyer'
    - foaf:firstName = 'John-Jules'

<foaf:Person@a43836633>
:   - foaf:name = 'Jeen Broekstra'
    - foaf:firstName = 'Jeen'

<foaf:Person@a85228505>
:   - foaf:name = 'Alexander Mädche'
    - foaf:firstName = 'Alexander'

<foaf:Person@a48552212>
:   - foaf:name = 'Björn Schnizler'
    - foaf:firstName = 'Björn'

<foaf:Person@a971541439>
:   - foaf:name = 'Alberto Trombetta'
    - foaf:firstName = 'Alberto'

<foaf:Person@a11090777>
:   - foaf:name = 'Christine Parent'
    - foaf:firstName = 'Christine'

<foaf:Person@a250331360>
:   - foaf:name = 'R. Schmidt'
    - foaf:firstName = 'R.'

<foaf:Person@a79573306>
:   - foaf:name = 'York Sure'
    - foaf:firstName = 'York'

<foaf:Person@a885257047>
:   - foaf:name = 'M. Punceva'
    - foaf:firstName = 'M.'

<foaf:Person@a74993404>
:   - foaf:name = 'I. V. Levenshtein'
    - foaf:firstName = 'I. V.'

<foaf:Person@a71003986>
:   - foaf:name = 'Steffen Staab'
    - foaf:firstName = 'Steffen'

<foaf:Person@a572406328>
:   - foaf:name = 'Frank Boer'
    - foaf:firstName = 'Frank'

<foaf:Person@a139477786>
:   - foaf:name = 'Maarten Menken'
    - foaf:firstName = 'Maarten'

<foaf:Person@a337716610>
:   - foaf:name = 'Manfred Hauswirth'
    - foaf:firstName = 'Manfred'

<foaf:Person@a086379337>
:   - foaf:name = 'Wiebe Hoek'
    - foaf:firstName = 'Wiebe'

<foaf:Person@a712561038>
:   - foaf:name = 'Marc Ehrig'
    - foaf:firstName = 'Marc'

<foaf:Person@a066600210>
:   - foaf:name = 'Danilo Montesi'
    - foaf:firstName = 'Danilo'

<foaf:Person@a093016135>
:   - foaf:name = 'Rogier Eijk'
    - foaf:firstName = 'Rogier'

<foaf:Person@a944339054>
:   - foaf:name = 'Frank van Harmelen'
    - foaf:firstName = 'Frank'

<foaf:Person@a98078619>
:   - foaf:name = 'Philippe Cudré-Mauroux'
    - foaf:firstName = 'Philippe'

<foaf:Person@a39510672>
:   - foaf:name = 'Z. Despotovic'
    - foaf:firstName = 'Z.'

<foaf:Person@a431956276>
:   - foaf:name = 'Stefano Spaccapietra'
    - foaf:firstName = 'Stefano'

<foaf:Person@a431956276b>
:   - foaf:name = 'Mike Papazoglou'
    - foaf:firstName = 'Mike'

<foaf:Person@a431956276c>
:   - foaf:name = 'Zahir Tari'
    - foaf:firstName = 'Zahir'

<foaf:Person@a70955601>
:   - foaf:name = 'A. Datta'
    - foaf:firstName = 'A.'

<foaf:Person@a467748807>
:   - foaf:name = 'Ateret Anaby-Tavor'
    - foaf:firstName = 'Ateret'

<foaf:Person@a3105947>
:   - foaf:name = 'Ronny Siebes'
    - foaf:firstName = 'Ronny'

<foaf:Person@a29105611>
:   - foaf:name = 'Karl Aberer'
    - foaf:firstName = 'Karl'

<foaf:Person@a958684218>
:   - foaf:name = 'Peter Mika'
    - foaf:firstName = 'Peter'

<foaf:Person@a94533498>
:   - foaf:name = 'Peter Haase'
    - foaf:firstName = 'Peter'

<foaf:Person@a900366022>
:   - foaf:name = 'Avigdor Gal'
    - foaf:firstName = 'Avigdor'

<qsdsquj@a246119474>
:   - foaf:name = 'Journal of Web Semantics'

<zauio@a131020767>


<qsdsquj@a70981683>


<zauio@a85849488>


<qsdsquj@a362042121>


<qsdsquj@a674639524>


<qsdsquj@a906774044>


<zqedzbx@spg04>


<zqedzbx@a72192307c>


<zqedzbx@a32071928c>


<zdqssqdb@a060097576>


<InProceedings@a64263824>


<InProceedings@a439508789>


<Article@a492378321>


<Article@a475526642>


<vccfsq@a71568377>


<zdqssqdb@a72192307>


<zdqssqdb@a32071928>


<Misc@a140583454>


<InProceedings@a11065952>


<Article@a80299267>


<dzajndsq@a108048723>


<Article@a456080390>


<Article@a846015923>

---

Generated by OWL2HTML
